# Perforated Patch Clamp Recordings in ex vivo Brain Slices from Adult Mice

**DOI:** 10.21769/BioProtoc.4741

**Published:** 2023-08-20

**Authors:** Simon Hess, Helmut Wratil, Peter Kloppenburg

**Affiliations:** Department of Biology, Institute for Zoology, Cologne Excellence Cluster in Aging Associated Diseases (CECAD), and Center of Molecular Medicine Cologne (CMMC), University of Cologne, Cologne, Germany

**Keywords:** Perforated patch clamp, Brain slices, Current clamp, Voltage clamp, Electrophysiology

## Abstract

Intracellular signaling pathways directly and indirectly regulate neuronal activity. In cellular electrophysiological measurements with sharp electrodes or whole-cell patch clamp recordings, there is a great risk that these signaling pathways are disturbed, significantly altering the electrophysiological properties of the measured neurons. Perforated-patch clamp recordings circumvent this issue, allowing long-term electrophysiological recordings with minimized impairment of the intracellular milieu. Based on previous studies, we describe a superstition-free protocol that can be used to routinely perform perforated patch clamp recordings for current and voltage measurements.

## Background

Neuronal activity is controlled to a large extent by intracellular signaling systems. This study describes how to perform perforated-patch clamp recordings for high-quality single-electrode whole-cell current and voltage clamp recordings with minimized impact on the cytosolic integrity.

Whole-cell patch clamp recordings have largely replaced intracellular recordings with sharp microelectrodes for single-electrode current and voltage clamp experiments. Because of the high seal resistance between the cell membrane and the recording electrode, this patch clamp configuration allows recording with a high signal-to-noise ratio, even from very small neurons. In addition, the whole-cell configuration provides low access resistance, which helps to ensure that the patch electrode solution can exchange freely with the cytoplasm. The whole-cell configuration is therefore also ideally suited to *control* the composition of the intracellular milieu, such as ion concentration, and to load the recorded neurons with tracers, sensors, and pharmacological agents. However, the free exchange of molecules between the cytoplasm and the patch electrode has a downside, as it impairs neuronal function by interfering with the cytosolic signaling system. Practically, this makes it impossible to use the whole-cell recording configuration for long-term measurements without significantly altering the physiological state of the recorded neurons.

The perforated-patch configuration, introduced initially by Lindau and Fernandez (1986) and Horn and Marty (1988), minimizes or even overcomes this drawback of the whole-cell configuration. Instead of rupturing the membrane under the recording electrode, which mediates the exchange between the cytosol and the electrode solution, pore-forming substances (ionophores) provide electrical access to the cell interior while largely maintaining the integrity of the cytoplasmic components of the neuron.

The *original* and most used perforating agents have been the antibiotic polyenes nystatin, amphotericin B, and the antibiotic polypeptide gramicidin (Horn and Marty, 1988; Akaike, 1994; Akaike and Harata, 1994; Kyrozis and Reichling, 1995; Klöckener et al., 2011; Könner et al., 2011; Hess et al., 2013). While polyenes and the peptide exhibit differences in their pore-forming mechanisms and ion selectivity (Myers and Haydon, 1972; de Kruijff and Demel, 1974; Russell et al., 1977; Tajima et al., 1996), their pores share key common properties: they are permeable to small molecules with a molecular weight up to ~200 Da, including monovalent ions (Urry, 1971; de Kruijff et al., 1974; Kyrozis and Reichling, 1995). However, they are neither permeable to divalent ions like Ca^2+^ nor intracellular signaling molecules of larger molecular weight. Building on previous studies, we describe here a protocol that can be used to routinely perform perforated-patch clamp recordings in brain slices of adult mice. The procedure is based on the use of amphotericin B as ionophore, as it has been, in our hands, the most suitable to achieve low access resistance and reproducibility. We describe how this approach can be used for current and voltage measurements and how this can be combined with single-cell labeling. We have applied this method to a variety of neuron types (see Table 1 for examples), but, for consistency, we only show data from substantia nigra pars compacta (SNpc) dopaminergic (DA) neurons here. In the context of DA neuron recording, one reviewer has strongly suggested mentioning the work of Cattaneo et al. (2021) describing cell-attached and whole-cell patch clamp recordings from DA neurons in the SNpc.

## Materials and reagents

**Animal preparation**

1. Dopaminergic neurons of the substantia nigra pars compacta (SNpc) from brain slices of adult (12–14 weeks old) mice
2. Isoflurane (AbbVie Deutschland GmbH and Co KG, catalog number: B506); storage: room temperature (RT)
3. Pattex Ultra Gel Matic (Henkel AG, EAN: 4015000444972)
4. Feather^®^ double edge blades (Plano, Feather, catalog number: 121-9)
5. Glycerol-based modified artificial cerebrospinal fluid (GACSF) (Ye et al., 2006) (see Recipes)
6. Artificial cerebrospinal fluid (ACSF) for current clamp recordings (see Recipes)
7. Artificial cerebrospinal fluid (ACSF) for Ca^2+^ current recordings in voltage clamp (see Recipes)

**Artificial cerebrospinal fluid and electrode solutions**

1. Calcium chloride 2-hydrate (CaCl_2_·2H_2_O), powder for analysis, ACS (AppliChem, catalog number: 131232); storage: RT
2. Potassium chloride (KCl) (Reag. USP) for analysis, ACS, ISO (AppliChem, catalog number: 131494); storage: RT
3. Magnesium chloride 6-hydrate (MgCl_2_·6H_2_O) (BP, Ph. Eur.) pure, pharma grade (AppliChem, catalog number: 141396); storage: RT
4. Sodium hydrogen carbonate (NaHCO_3_) (Reag. USP) for analysis, ACS, ISO (AppliChem, catalog number: 131638); storage: RT
5. Sodium chloride (NaCl) for analysis, ACS, ISO (AppliChem, catalog number: 131659); storage: RT
6. Sodium di-hydrogen phosphate 1-hydrate (NaH_2_PO_4_·H_2_O) (Reag. USP, Ph. Eur.) for analysis, ACS (AppliChem, catalog number: 131965); storage: RT
7. Sodium hypochlorite 14% Cl_2_ (ClNaO) in aqueous solution (VWR, catalog number: 27900); storage: RT
8. Sodium hydroxide (NaOH) 1 mol/L (1 N) in aqueous solution (VWR, catalog number: 31627); storage: RT
9. D(+)-glucose anhydrous BioChemika (AppliChem, catalog number: A1422); storage: RT
10. HEPES for molecular biology (AppliChem, catalog number: A3724); storage: RT
11. Glycerol bioreagent, suitable for (insect) cell culture, suitable for electrophoresis, ≥ 99% (GC) (Sigma-Aldrich, catalog number: G2025); storage: RT
12. C_6_H_11_KO_7, _potassium-D-gluconate (Sigma-Aldrich, catalog number: G4500); storage: RT
13. CsCl, cesium chloride (Sigma-Aldrich, catalog number: C4036); storage: RT
14. CsOH, cesium hydroxide 99.9% (Sigma-Aldrich, catalog number: 232041); storage: RT
15. EGTA, ethylene glycol-bis(2-aminoethylether)-N,N,N′,N′-tetraacetic acid (Sigma-Aldrich, catalog number: 03777-10G)
16. TEA, Tetraethylammonium chloride (Sigma-Aldrich, catalog number: T2265); storage: RT
17. Picrotoxin ≥ 97.5% (Roth, catalog number: 7093.1); storage: RT
18. CNQX, 6-Cyano-7-nitrochinoxalin-2,3-dion ≥ 98% (HPLC), solid (Sigma-Aldrich, catalog number: C127); storage: RT
19. DAP-5, DL-2-amino-5-phosphopentanoic acid solid (Sigma-Aldrich, catalog number: A5282); storage: RT
20. TTX, tetrodotoxin citrate (Biotrend, catalog number: ARCD-0640-1); storage: 4 °C
21. Tetramethylrhodamine-dextran, 3000 MW, anionic, lysine fixable (Thermo Fisher Scientific, Invitrogen, catalog number: D3308); storage: -20 °C
22. Biocytin (Sigma-Aldrich, catalog number: B4261); storage: -20 °C
23. Electrode solution (current clamp) (see Recipes)
24. Electrode solution (voltage clamp: Ca^2+^ currents) (see Recipes)

**Ionophore preparation and patch clamp recordings**

1. Dimethyl sulfoxide (DMSO) (Sigma-Aldrich, catalog number: D8418); storage: RT
2. Amphotericin B from *Streptomyces* sp. ~80% (HPLC), powder (Sigma-Aldrich, catalog number: A4888); storage: 4 °C
3. Microtubes, PP, 1.5 mL, with attached cap, BIO-CERT^®^ PCR QUALITY (Brand, catalog number: 780420)
4. Silver wire for electrodes (Science Products, catalog number: AG-10W)
5. Amphotericin B stock solution (see Recipes)

## Equipment

1. Pipette puller (Narishige, model: PC-10)
2. Electrode borosilicate glass (Science Products, catalog number: GB150-8P)
3. Micromanipulator (Luigs-Neumann, model: mini 23)
4. Data acquisition unit [Cambridge Electronic Design Ltd. (CED), model: Micro 1401 MkII]
5. Patch clamp amplifier (HEKA, model: EPC-10 USB)
6. Temperature controller (Warner Instrument Corp., model: TC-324B)
7. Inline heater (Warner Instrument Corp., model: SH-27B)
8. Fixed stage upright microscope (Olympus, model: BX51WI) equipped with a 20× water-immersion objective (XLUMPLFL, 0.95 numerical aperture, 2 mm working distance, Olympus), a 4× magnification changer (U-TVAC, Olympus), infrared differential interference contrast optics (Dodt and Zieglgänsberger, 1990), and fluorescence optics
9. Vibratome (Leica, model: VT1200S)
10. Heating circulator (Julabo, model: ED)
11. Anesthesia machine (Groppler Medizintechnik, Univet, model: Porta)
12. Magnetic stirrer (VWR, catalog number: 12365-428)
13. Vortex mixer (Scientific Industries, Vortex Genie 2, model: SI-0256)
14. Ultrasonic bath (Bandelin Electronic, model: DT 31 H)
15. Centrifuge (Eppendorf AG, model: 5415 D)
16. Peristaltic pump (Ismatec, model: ISM597D) with Tygon tube (1.6 and 2.4 inner diameters, Ismatec, Tygon, catalog number: T3350)
17. Fine scissors, sharp (FST, catalog number: 14060-09)
18. Surgical scissors, blunt (FST, catalog number: 14001-14)
19. Double spatulas, micro (VWR, catalog number: 231-2261)
20. Slice anchor (Warner Instruments, model: SHD-26H/15)

## Software

1. PatchMaster (HEKA, Ver. 2.32, www.heka.com)
2. Spike2 (Cambridge Electronic Design Ltd. (CED), www.ced.co.uk)

## Procedure

- **Animals and preparation of mature/adult brain slices**
  1. Lightly anesthetize the animal with isoflurane (5%).
  2. Decapitate the mouse with surgical scissors.
  3. Insert the fine sharp scissors into the spinal cord canal and make an incision by cutting along the midline of the skull from caudal to rostral to approximately the frontonasal suture.
  4. To open the skull, grab one side of the skull with the groove of the tissue forceps and rotate the forceps 180 degrees. Repeat the procedure with the other side of the skull.
  5. Remove the brain with a spatula: insert the spatula between the olfactory bulb and the rest of the brain and pull the mouse brain out.
*Note: Be as quick as possible (≤ 30 s), especially when dissecting older animals!*
  6. Make two trim cuts (caudal, rostral) with a standard razor blade.
  7. Glue the brain onto the vibratome plate using Pattex Ultra Gel Matic.
  8. Cut the brain with the vibratome under 4 °C cold carbogenated (Carbogen, 95% O_2_, 5% CO_2_) GACSF (see Recipes) using a Feather^®^ razor blade.
  9. Vibratome settings: speed 0.06 mm/s; amplitude 1.05 mm; thickness 290 μm.
  10. Incubate the brain slices for 25–50 min in a recovery bath (carbogenated ACSF, see Recipes) at 37 °C.
  11. Keep the brain slices in carbogenated ACSF at RT. The slices should be used within 5 h.
  12. Place the brain slices in the recording chamber for the recordings held down with a slice anchor and superfused with carbogenated ACSF at the desired experimental temperature.
- **Preparation and usage of the ionophore (amphotericin B)**
  1. Preparation
    - Make a stock solution of amphotericin B by dissolving 4 mg of amphotericin B in 100 μL of DMSO.
    - Sonicate the stock solution until the solution becomes a uniformly turbid yellowish solution.
    - Vortex the stock solution several times.
    - Prepare two 1.5 mL microtubes, each filled with 1 mL of electrode solution (for current or voltage clamp). A defined volume of amphotericin B stock solution (see Table 1) is added to 1 mL of electrode solution, while the solution in the other microtube remains free of amphotericin (*tip fill*). In our experience, a final amphotericin concentration of 160–200 μg/mL (4–5 μL stock solution) ensures excellent perforation and recording conditions for many neuron types. However, depending on cell type and recording mode (current clamp or voltage clamp), optimizing the amphotericin concentration might be useful or even necessary (for examples, see Table 1).
      **Table 1.** Recommended amphotericin B concentrations for different neuron types

Recording mode	Neuron type	Amount of stock solution (μL)	Final amphotericin B concentration (μg/mL)
Current clamp	Dopaminergic (DA) neuron (midbrain) (Hess et al., 2013)	4.5	180
	Serotonergic neuron (dorsal raphe nucleus) (Xiao et al., 2021)	4	160
	POMC neuron (hypothalamus) (Paeger et al., 2017)	4	160
	AgRP neuron (hypothalamus) (Steculorum et al., 2016)	4	160
	V2a neurons (spinal cord) (Reinoss et al., 2020)	20	800
Voltage clamp	Dopaminergic (DA) neuron (midbrain) (Siller et al., 2022)	10	400
    - Shake (by hand) the amphotericin B–containing electrode solution several times.
    - Add 1% (0.1 mg/100 μL) tetramethylrhodamine-dextran to the amphotericin B–containing electrode solution. Tetramethylrhodamine-dextran is used to check the integrity of the cell membrane once the ionophore has perforated the membrane (for further explanation, see below.)
    - Put all three solutions (stock, tip fill, and amphotericin B–containing electrode solution) on ice. The amphotericin B–containing electrode solution can be used for up to three hours. After that, prepare a fresh solution.
- **Setting up the perforated-patch clamp recording for current clamp**
  1. Pull electrodes with resistances between 3 and 5 MΩ.
  2. Fill the electrode tips with plain electrode solution (tip fill without amphotericin B).
  3. For the backfill, use the amphotericin B–containing electrode solution.
  4. Put the electrode in the bath but do not apply positive pressure!
*Note: Commonly, in patch clamp recordings from brain slices, positive pressure is applied when the electrode is advanced to the cell to prevent clogging of the electrode tip and to clean the target cell. However, in this case, the amphotericin B–containing solution would be driven into the electrode tip, severely compromising seal formation.*
  5. Only when the electrode is positioned directly in front of the cell, apply a slight positive pressure with the mouthpiece and continue to approach the cell. As soon as a *dent* forms in the cell membrane in front of the electrode, immediately release the positive pressure (Figure 1C).
  6. Apply gentle negative pressure with the mouthpiece and constantly monitor the test pulse (voltage clamp mode, holding potential: 0 mV, test pulse amplitude: 5 mV) and the seal resistance.
  7. Release the pressure after you have reached a seal resistance of > 600 MΩ.
    *Note: Sometimes, you will not be able to reach a* giga seal *since the ionophore already starts perforating the cell membrane during the sealing process, which can take up to 20 min.*
  8. Switch to *current clamp* and monitor the perforation process. Ideally, you should see a continuous transition from the *on cell* to the *perforated* configuration, as shown in Figure 1A.
    **Figure 1.**
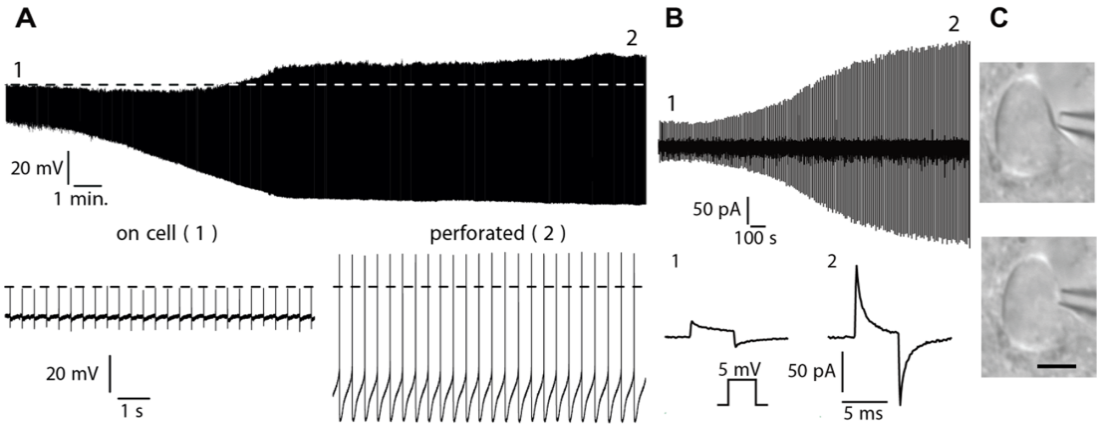
Perforation process. Original recordings showing the transition from the *on-cell* to the *perforated-patch* recording configuration under current clamp (A) and voltage clamp (B). Bottom: segments of the original traces shown in the top panel in higher time resolution. The numbers indicate the times from which the segments originate. (B). Voltage pulses (5 mV, 5 ms, HP = -60 mV) were applied every 10 s. Bottom left: test pulse before perforation (*R*_S_ > 100 MΩ), Bottom right: test pulse after perforation has reached a steady state (*R*_S_ < 20 MΩ). HP, holding potential. (C). Seal formation. Top: slight positive pressure forms a dent in the cell membrane in front of the electrode. Bottom: after releasing the positive pressure, negative pressure supports seal formation. Scale bar: 10 μm.
    The perforation process is reflected by an increase in action potential amplitude, indicating a decrease in series resistance. During this process, spontaneous conversions to the whole-cell configuration can occur, which become evident by an abrupt increase in action potential amplitude (Figure 2A). In many cases, this conversion is accompanied by hyperpolarization and the cessation of spiking activity (e.g., due to the opening of K_ATP_ channels).If no further changes in the parameters mentioned above occur, the perforation process is complete, and the actual measurements can begin.
*Note: To get meaningful measurements of electrophysiological properties of the investigated cell, the series resistance should ideally be well below 60 MΩ.*
    **Figure 2.**
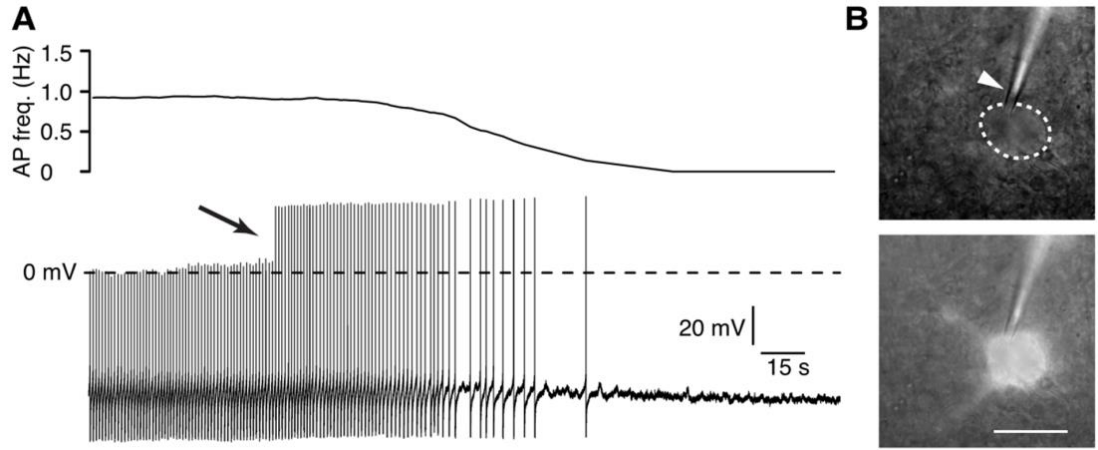
Tetramethylrhodamine-dextran as a marker for rupture of the membrane. Spontaneous conversion from the perforated patch to the whole-cell configuration. (A). Current clamp recording with corresponding frequency plot showing the spontaneous rupture of the membrane patch during the perforation process. The arrow marks the jump in spike amplitude due to the rupture of the membrane. (B). Tetramethylrhodamine-dextran fluorescence is confined to the tip of the electrode, indicating the integrity of the membrane patch (top). After rupturing the membrane, tetramethylrhodamine-dextran has diffused into the neuron (bottom). Scale bar: 20 μm.
  9. During the recording, check the tetramethylrhodamine-dextran fluorescence regularly to see if the membrane patch in the electrode is still intact (Figure 2B). If not, terminate the recording.
    Adding tetramethylrhodamine-dextran helps significantly to monitor the integrity of the perforated patch. This is very useful since spontaneous ruptures of the membrane patch are often not immediately reflected in noticeable changes in the action potential amplitude and the series resistance once the membrane has been perforated. Due to its molecular weight of 3,000 Da, tetramethylrhodamine-dextran can neither permeate through the cell membrane nor the pores formed by the ionophores, making it possible to detect whether the membrane patch has been ruptured.
*Note: In our experience, tetramethylrhodamine-dextran does not compromise the quality of the recording. Although other fluorescent molecules may be suitable, checking whether they interfere with the recording is crucial.*
    Once the perforated configuration is established, long-term recordings can be performed with a significantly minimized rundown compared to the whole-cell configuration. Figures 3–5 show examples of perforated-patch clamp recordings in different experimental settings, illustrating the possibilities of this recording configuration, e.g., in long-lasting current and voltage clamp experiments. A direct comparison between the whole-cell and perforated configurations is shown in Figure 3. In the whole-cell configuration, action potential frequency decreased dramatically within the first 10 min (Figure 3A). In contrast, the frequency remained stable in the perforated-patch clamp recordings (Figure 3B). This configuration is, therefore, particularly suitable for long-term (> 2 h) pharmacological experiments where it is desirable to demonstrate reversibility and reproducibility in the same recording. Such an experiment is shown in Figure 4, where cocaine was bath-applied and washed out twice.
    **Figure 3.**
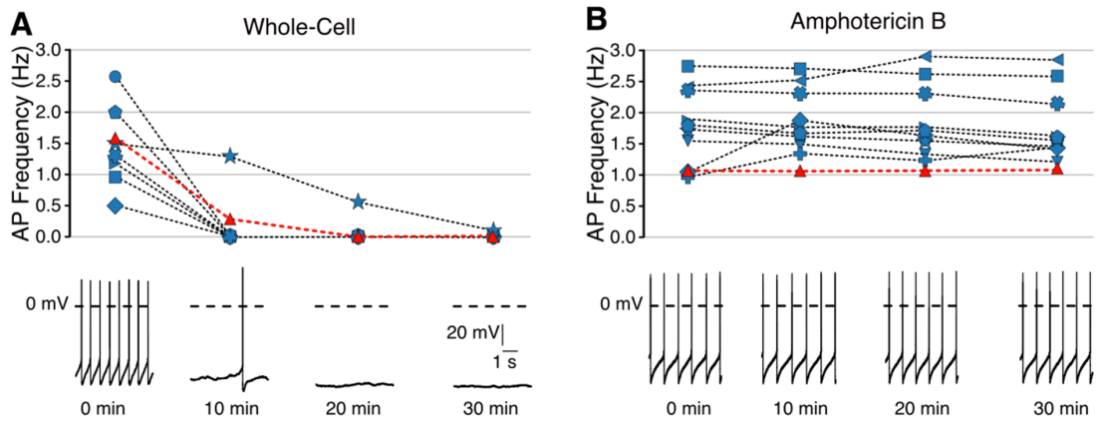
Time course of spontaneous action potential frequency from 30 min measurements in whole-cell (A) and perforated-patch recordings (B). Whole-cell recordings (n = 9); perforated-patch recordings (n = 11). The traces at the bottom correspond to the red symbols in the respective frequency plots.
    **Figure 4.**
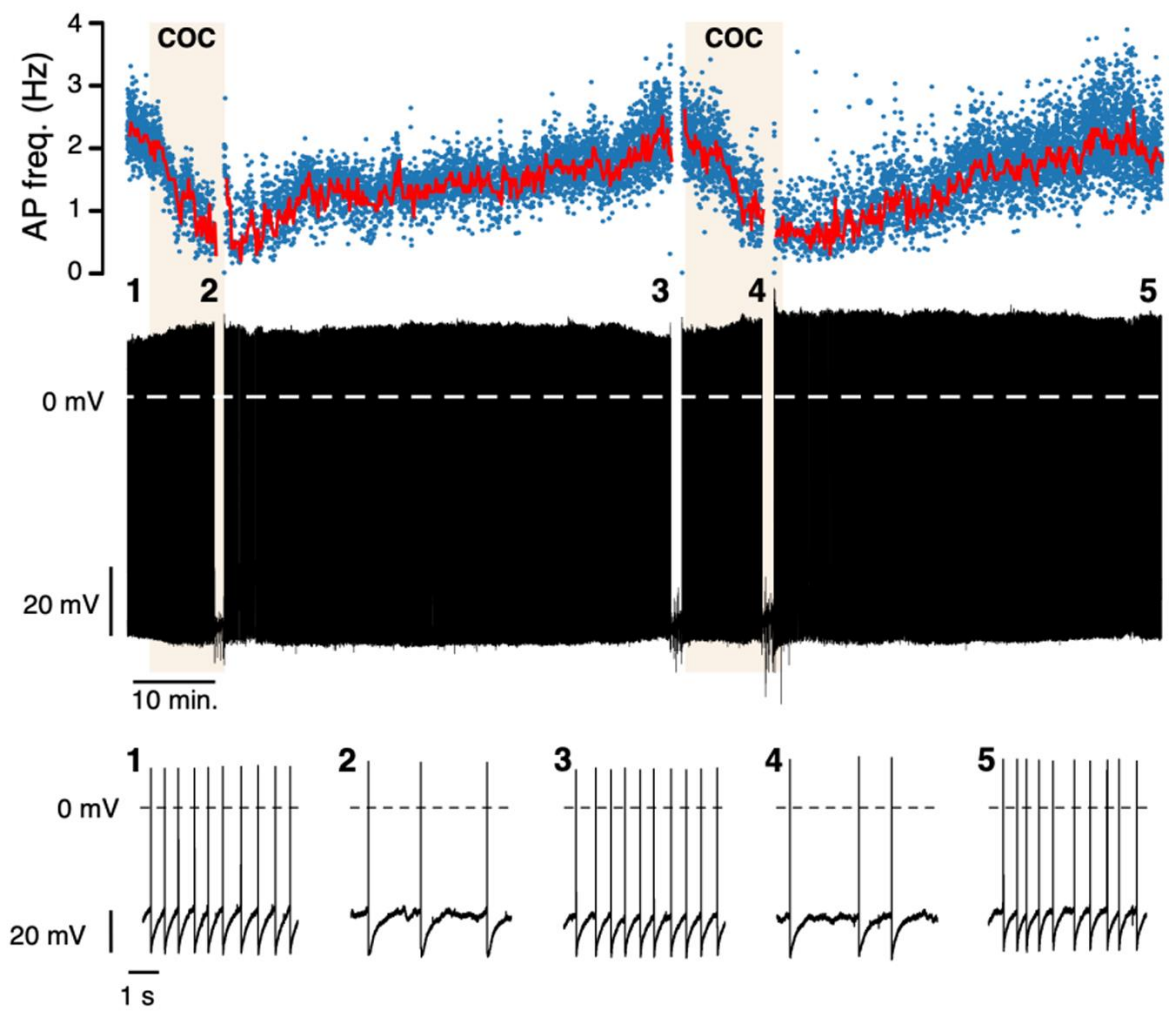
Long-term perforated patch clamp recording. Example of a neuropharmacological experiment (~2 h) using the perforated patch configuration. Top: action potential frequency (Hz) of a mouse dopaminergic (DA) substantia nigra pars compacta (SNpc) neurons during two exposures to 10 μM cocaine (COC). Blue dots indicate the instantaneous frequency, and the red line indicates the average frequency (bin size: 10 s). Middle: original recording. The numbers correspond to the sections displayed at the bottom. Current pulses were applied at the sections where gaps in the recordings occurred.
- **Setting up the perforated patch clamp recording for voltage clamp**
  To perform voltage clamp experiments, follow steps 1–7 of section C and then continue with the following steps.
*Note: Higher amphotericin B concentrations might be useful (see Table 1).*
  1. Switch the holding potential to -60 mV.
  2. Continue applying 5 mV test pulses in voltage-clamp mode (Figure 1B).
  3. Wait until the perforation has reached a steady state (stable current amplitude).
  4. Check the integrity of the membrane patch occasionally, as already described.
    Examples of successful voltage clamp recordings are given in Figure 5.
    **Figure 5.**
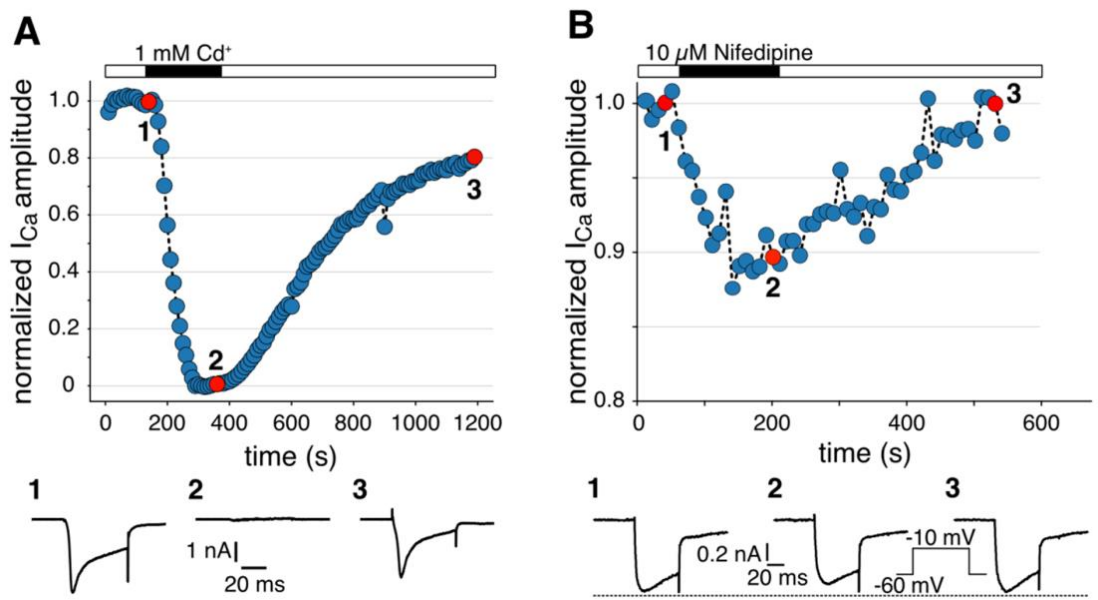
Perforated patch voltage clamp recordings of voltage-activated Ca^2+^ currents (*I*_Ca_). *I*_Ca_ was induced by depolarizing voltage steps to 0 mV (A) or -10 mV (B) from a holding potential of -60 mV every 10 s. A, B. Example of peak *I*_Ca_ in a mouse dopaminergic (DA) substantia nigra pars compacta (SNpc) neuron plotted over time during cadmium (A; 1 mM) or nifedipine (B; 10 μM) bath application. The numbers correspond to calcium current traces shown at the bottom.
- **Complementary application: single-cell labeling**
  1. Use an electrode solution containing a tracer (e.g., biocytin).
  2. Perform the perforated-patch clamp experiment as described.
  3. After completion of the electrophysiological experiment, rupture the membrane patch either by gentle suction or by a large hyperpolarizing voltage pulse (~1–1.5 V) to allow cell labeling by diffusion of the tracer into the cell.
  4. Further processing of the brain slice containing the labeled neuron can be performed by using standard histological and immunohistochemical procedures.
    An example of successful labeling of a recorded neuron is given in Figure 6.
    **Figure 6.**
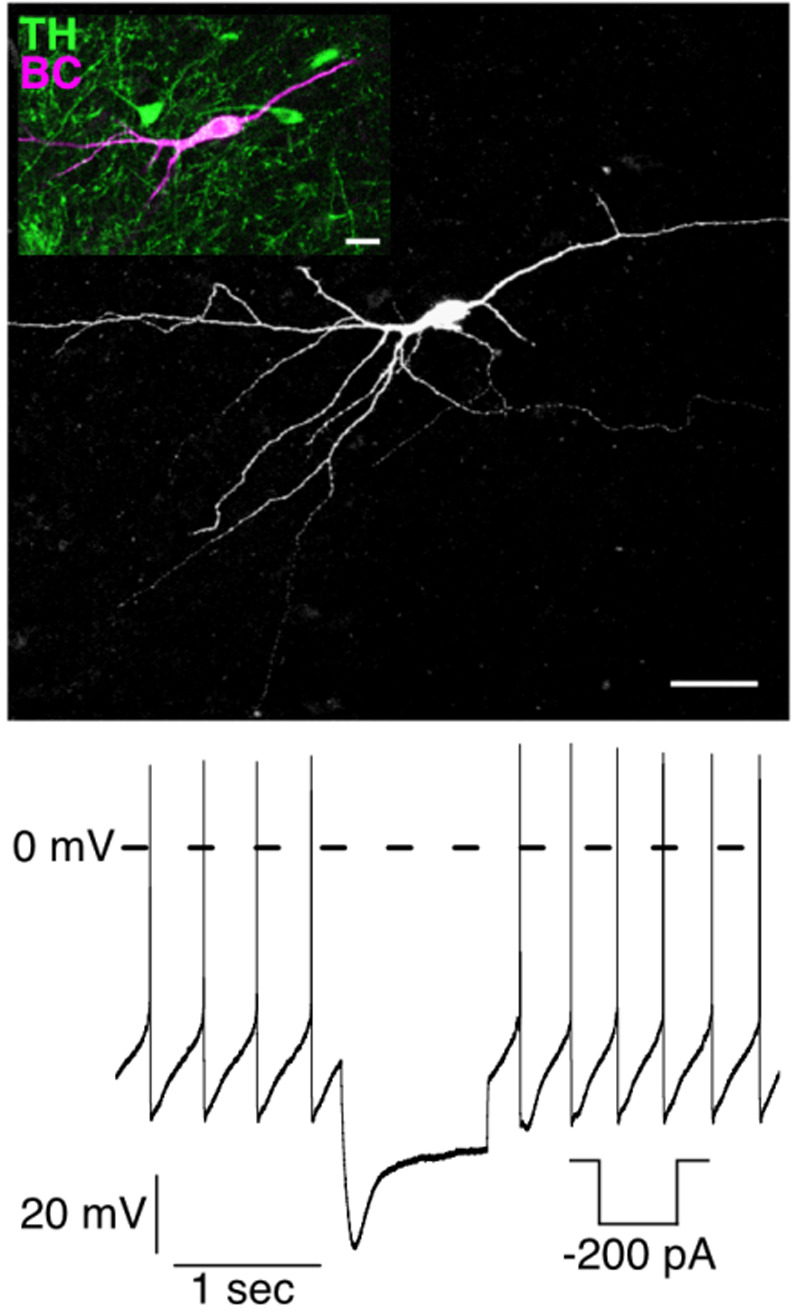
Morphological and immunohistochemical characterization. Post-recording immunohistochemical identification of a dopaminergic (DA) neuron filled with biocytin after the electrophysiological experiment (current clamp). Top: single-cell staining of the DA neuron with biocytin-streptavidin. Scale bar: 50 μm. Inset: immunohistochemical co-labeling against tyrosine-hydroxylase (TH), a marker for DA neurons. Scale bar: 20 μm. Bottom: perforated-patch clamp recording (current clamp) of the labeled neuron showing pacemaker activity and a prominent ‘sag’ potential during hyperpolarization, both characteristic features of DA substantia nigra pars compacta (SNpc) neurons.

## Data analysis

Data were recorded with the PatchMaster software (HEKA). Data were sampled at 10 kHz and low-pass filtered at 2 kHz with a four-pole Bessel filter. Data were analyzed with Spike2 and custom-made scripts written in Python 3.x.

## Recipes

In the following recipes, the measured osmolarity is given. When composing solutions, we recommend that the osmolarity of the electrode solution (intracellular solution) be 10–20 mOsmol·L^-1^ lower than the extracellular solution.

**Animal preparation**

1. **Glycerol-based modified artificial cerebrospinal fluid (GACSF)**
  225 mM glycerol
  2.5 mM KCl
  2 mM MgCl_2_·6H_2_O
  2 mM CaCl_2_·2H_2_O
  1.2 mM NaH_2_PO_4_
  21 mM NaHCO_3_
  10 mM HEPES
  5 mM glucose
  Gassed with carbogen and adjusted to pH = 7.2 with NaOH, resulting in an osmolarity of 300–310 mOsmol/L.
2. **Artificial cerebrospinal fluid (ACSF) for current clamp recordings**
  125 mM NaCl
  2.5 mM KCl
  2 mM MgCl_2_·6H_2_O
  2 mM CaCl_2_·2H_2_O
  1.2 mM NaH_2_PO_4_
  21 mM NaHCO_3_
  10 mM HEPES
  5 mM glucose
  Gassed with carbogen and adjusted to pH = 7.2 with NaOH, resulting in an osmolarity of ~310 mOsmol/L.
*Note: Block of glutamatergic and GABAergic synaptic input can be achieved by adding 0.1 mM picrotoxin, 0.05 mM DAP-5, and 0.01 mM CNQX to the ACSF.*
3. **Artificial cerebrospinal fluid (ACSF) for Ca^2+^ current recordings in voltage clamp**
  66.5 mM NaCl
  20 mM CsCl
  40 mM TEA-Cl
  4 mM MgCl_2_·6H_2_O
  3 mM CaCl_2_·2H_2_O
  21 mM NaHCO_3_
  10 mM HEPES
  5 mM Glucose
  0.001 mM TTX
  0.1 mM picrotoxin
  0.05 mM DAP-5
  0.01 mM CNQX
  Gassed with carbogen and adjusted to pH = 7.2 with HCl, resulting in an osmolarity of ~305 mOsmol/L.

**Patch clamp recordings**

1. **Electrode solution (current clamp)**
  141 mM K-gluconate
  10 mM KCl
  10 mM HEPES
  0.1 mM EGTA
  2 mM MgCl_2_·6H_2_O
  Adjusted to pH = 7.2 with KOH, resulting in an osmolarity of ~290 mOsmol/L.
2. **Electrode solution (voltage clamp: Ca^2+^ currents)**
  146 mM CsCl
  10 mM HEPES
  0.1 mM EGTA
  2 mM MgCl_2_·6H_2_O
  Adjusted to pH=7.2 with CsOH, resulting in an osmolarity of ~290 mOsmol/L.

**Ionophore (amphotericin B) solutions**

1. **Stock solution**
  100 μL of DMSO
  4 mg of amphotericin B
